# Comparing Feto-Maternal Outcomes in Pregnant Women With Normal and Abnormal Liver Function Tests: A Prospective Observational Study

**DOI:** 10.7759/cureus.56811

**Published:** 2024-03-24

**Authors:** Jyotsna Pathak, Neeru Goel, Sanjeev Kumar Jha, Sweety Rani, Kanchan Kumari, Ranjana Ranjana

**Affiliations:** 1 Obstetrics and Gynecology, Indira Gandhi Institute of Medical Sciences (IGIMS), Patna, IND; 2 Gastroenterology, Indira Gandhi Institute of Medical Sciences (IGIMS), Patna, IND

**Keywords:** fetal outcome, maternal outcome, pregnancy, normal liver function test, abnormal liver function test

## Abstract

Introduction: Pregnant women with abnormal liver function tests (LFTs) require proper evaluation and timely management to reduce maternal and fetal morbidity and mortality.

Objective: The present study was done with the objective of determining feto-maternal outcomes in antenatal women with abnormal LFTs and comparing them with antenatal women having normal liver function. The prevalence and possible causes of derangements in LFT were also identified.

Method: Pregnant women referred to an antenatal clinic for several reasons pertaining to abnormal liver functions, and those admitted to the labor room for delivery with abnormal LFTs were included in the study. The pregnant women with abnormal LFT were studied prospectively, and they were compared with pregnant women having normal LFT. The fetal and maternal outcomes were also noted.

Results: The pregnant women attending the antenatal clinic with a history of pruritus, abdominal pain, jaundice, nausea/vomiting, hypertension ascites, etc. and delivered at our facility were evaluated. One hundred and eight women had abnormal LFT defined by criteria laid down in material and methods. Eighty-seven women with normal LFT were taken for comparison. In the abnormal LFT, the main cause was intrahepatic cholestasis of pregnancy (IHCP). There were 6 (5.5%) maternal deaths in this group and none in the normal LFTs. There were 6 (5.6%) fetal deaths and 4 (4.6%) in the other group (p-value=1). The prevalence of abnormal LFT was 9.11% throughout pregnancy. Increased bilirubin and alkaline phosphatase (ALP) were significantly correlated with maternal mortality, while gestational age at birth, presence of meconium, appearance, pulse, grimace, activity, and respiration (APGAR) score, maternal mortality, and raised alkaline phosphatase level were found to be significantly associated with fetal mortality.

Conclusion: Patients with abnormal LFT were significantly associated with maternal morbidity and mortality. However, fetal outcomes in patients with abnormal and normal LFT were similar. Hyperbilirubinemia and raised alanine aminotransferase (ALT) were significant predictors of maternal mortality.

## Introduction

Liver function tests (LFTs) during pregnancy are comparable to those of non-pregnant women, with the exception of serum alkaline phosphatase (ALP) levels, which may reach two to four times the normal adult upper reference value [1]. LFTs include transaminases, aspartate aminotransferase (AST), alanine aminotransferase (ALT), ALP, bilirubin, and serum albumin. ALT and AST are both markers of hepatocellular injury; ALP, bilirubin, and bile acid are suggestive of cholestasis; and albumin and the international normalized ratio (INR) reflect the synthetic functions of the liver. The incidence of abnormal LFTs during pregnancy varies between 0.72% and 6.7% [2,3].

Causes of abnormalities in LFT during pregnancy can be divided into three major groups. The first group is liver disorders specific to pregnancy, including hyperemesis gravidarum, pre-eclampsia, hemolysis, elevated liver enzyme and low platelets (HELLP) syndrome, acute fatty liver of pregnancy (AFLP), and intrahepatic cholestasis of pregnancy (IHCP), all of which are mostly trimester-specific. The second group includes intercurrent liver diseases during pregnancy, such as viral hepatitis, sepsis, and cholelithiasis. The third group includes pregnancy with pre-existing liver diseases, such as chronic hepatitis, cirrhosis of the liver, and Budd-Chiari syndrome [3]. Abnormal LFTs can have a significant impact in pregnancy, leading to adverse maternal and neonatal outcomes. Increased incidence of pruritus, kidney injury, coagulopathy, infection, abortion, preterm birth, meconium staining of amniotic fluid, low birth weight, and intrauterine deaths have been seen in pregnant women with abnormal LFTs [4]. Maternal mortality in patients with hepatic dysfunction ranges from 1.37% to 39.3%. Similarly, perinatal mortality in patients with abnormal LFTs ranges from 16% to 38% [5]. Such patients require proper evaluation and timely management to reduce maternal and fetal morbidity and mortality. In India, most of the studies addressing the issues of abnormal LFTs take place in northern and southern India, with little data available from eastern India.

The primary objective of the study was to determine feto-maternal outcomes in antenatal women with abnormal LFTs and to compare them with antenatal women with normal liver function. The secondary objectives were to estimate the prevalence of abnormal LFTs in pregnant women and to identify the possible causes of abnormal LFTs in antenatal women.

## Materials and methods

This prospective observational study was carried out in a tertiary care center in the eastern part of India from March 2021 to July 2023. The study protocol conformed to the ethical guidelines of the 1975 Declaration of Helsinki. Institutional Ethics Committee approved this study (38/IEC/IGIMS/2021). Written consent was obtained from all patients before including them in the study.

Pregnant women referred to the antenatal clinic for several reasons pertaining to abnormal liver function, and those admitted in the labor room for delivery with the abnormal LFT were included in the study. The main indications for doing LFTs in the antenatal clinic were pruritus, abdominal pain, jaundice, nausea/vomiting, hypertension, ascites, and clinical evidence of infection, whereas LFTs were routinely performed for patients admitted to the labor and delivery departments. Pregnant women with normal LFTs were taken as controls.

The biochemistry department of our center defined abnormal LFTs as values higher than the normal reference value range. An ALP value was considered abnormal if it was twice the upper normal limit (UNL) range. Normal LFTs were designated as follows: total serum bilirubin of 0.2-1.0 mg/dL, direct bilirubin<0.2 mg/dL, indirect bilirubin of 0.2-0.8 mg/dL, AST=5-40 IU/L, ALT=5-45 IU/L, ALP≤120 U/L, total protein=6-8 g/dL, and albumin=3.5-5.5 g/dL.

Patients were excluded from the study if they met the following criteria: those with poorly controlled diabetes mellitus (HbA1c>8%), serum creatinine >1.5 mg/dL, malignancy, acquired immunodeficiency disease, decompensated heart disease (New York Heart Association (NYHA) grades 3 and 4), poorly controlled pre-existing hypertension, chronic obstructive airway disease or other systemic severe comorbidities, and COVID-19-positive patients.

A detailed clinical assessment was done for each patient enrolled in the study, which included menstrual and obstetrical histories. Routine investigations were also done for each patient, including complete blood count, blood sugar with glucose challenge test, hepatitis B surface antigen (HBsAg), anti-hepatitis C virus (anti-HCV), human immunodeficiency virus (HIV), blood group, LFT, kidney function test, urine analysis, and ultrasound of the whole abdomen with assessment of fetal well-being. Investigations such as serum bile acid, INR, activated partial thromboplastin time (APTT), peripheral blood smear, immunoglobulin M (IgM) antibody against hepatitis A and hepatitis E viruses, non-stress test (NST), color doppler of the spleno-portal axis, and fundoscopy were done as and when required. Periodic monitoring of liver functions was done throughout pregnancy and up to one week after delivery. Babies were evaluated at birth and followed for seven days after delivery.

Hyperemesis gravidarum was diagnosed if patients presented with persistent vomiting in the first trimester requiring hospitalization and associated with dehydration and electrolyte imbalance. Pre-eclampsia was diagnosed in cases of systolic blood pressure of ≥140 mmHg developing after 20 weeks of gestation measured on two occasions, 4-6 h apart, with one or more of the following associated features: (a) symptoms of pre-eclampsia (e.g., severe headache, visual disturbance, epigastric pain, and vomiting); (b) proteinuria>300 mg/24 h, 1+on dipstick (0.3 gm/L), a urine protein/creatinine ratio of >0.3 on two random samples collected 4-6 h apart and/or evidence of end-organ damage. Pregnancies with pre-eclampsia were managed according to American College of Obstetrics and Gynecology guidelines.

HELLP syndrome was diagnosed using the Tennessee criteria, which included hemolysis, abnormal peripheral blood smear (burr cells, schistocytes), elevated bilirubin≥1.2 g/dL, low serum haptoglobin, increased LDH≥2 × UNL (>600 U/L), elevated liver enzymes (AST, ALT≥2 × UNL (≥72 IU/L), and low platelet count (≤100,000/mm^3^) [6]. AFLP was diagnosed using the Swansea criteria, which included the presence of six or more of the following features in the absence of another explanation: (1) ascites or bright liver on ultrasound, (2) coagulopathy (PT>14 s or APTT>34 s), (3) elevated serum ammonia levels (>47 µmol/L), (4) elevated serum transaminases (>42 IU/L), (5) elevated serum bilirubin (>0.8 mg/dL), (6) elevated serum uric acid level (>5.7 mg/dL), (7) encephalopathy, (8) hypoglycemia (<72 mg/dL), (9) leukocytosis (>11,000/mm^3^), (10) creatinine (>1.7 mg/dL), and (11) microvesicular steatosis on liver biopsy [7]. Pregnancies with HELLP and AFLP were treated in the intensive care unit in collaboration with an intensivist, and delivery was expedited. IHCP was diagnosed if the patient developed pruritus in the second- or third-trimester of pregnancy that improved soon after delivery with elevated levels of serum bile acids (>10 µmol/L) after exclusion of other causes [6].

Patients with hepatitis B received antiviral treatment if required. Neonates born to HBsAg-positive mothers received active and passive immunization of hepatitis B as per recommendations. Patients with hepatitis E were treated with supportive management. Patients with liver disease were monitored for the development of complications such as gastrointestinal bleeds, ascites, encephalopathy, and electrolyte abnormalities.

The primary objective of our study was to determine maternal and fetal mortality. Maternal mortality was defined as the death of the woman while pregnant or within six weeks of termination of pregnancy. Fetal death was defined as the death of the fetus before or during delivery or within seven days of delivery [8]. Whether labor was spontaneous or induced was also observed, as well as a mode of delivery. Maternal morbidity in terms of postpartum hemorrhage, need for blood transfusion, intensive care unit admission and reason for admission, and total duration of hospital stay were noted. The following fetal parameters were observed: appearance, pulse, grimace, activity, and respiration (APGAR) score, presence of meconium, and neonatal intensive care unit (NICU) admission.

Sample size calculation

According to the Ministry of Health and Family Welfare Department of India during 2016-2018, the maternal mortality rate in India was 0.11% [9]. Many studies have reported different maternal mortality rates due to abnormal LFTs, with a mean value of approximately 12.6% [10-12]. Assuming an alpha error of 5%, a study power of 80%, and a lost-to-follow-up rate of 10%, the necessary sample size was calculated to be 59 patients in each arm of the study.

Statistical analysis

Baseline characteristics were expressed as mean ± standard deviation (SD) for quantitative variables with a normal distribution or medians (ranges) for quantitative variables with a non-normal distribution. The comparison of quantitative parameters was done using the Student's independent t-test or the Mann-Whitney U test whenever applicable. Qualitative variables were expressed in numbers or percentages and were compared using the chi-squared test or Fisher’s exact test. The level of significance was expressed in terms of the p-value, where p<0.05 is considered statistically significant. Bivariate analysis was used to determine factors associated with maternal or fetal mortality. Factors found significant in bivariate analysis were analyzed, with multivariate analysis using stepwise logistic regression. Statistical analysis was done using Statistical Package for the Social Sciences version 23.0 (SPSS Inc., Chicago, IL, USA).

## Results

Demographic profile

**Figure 1:**
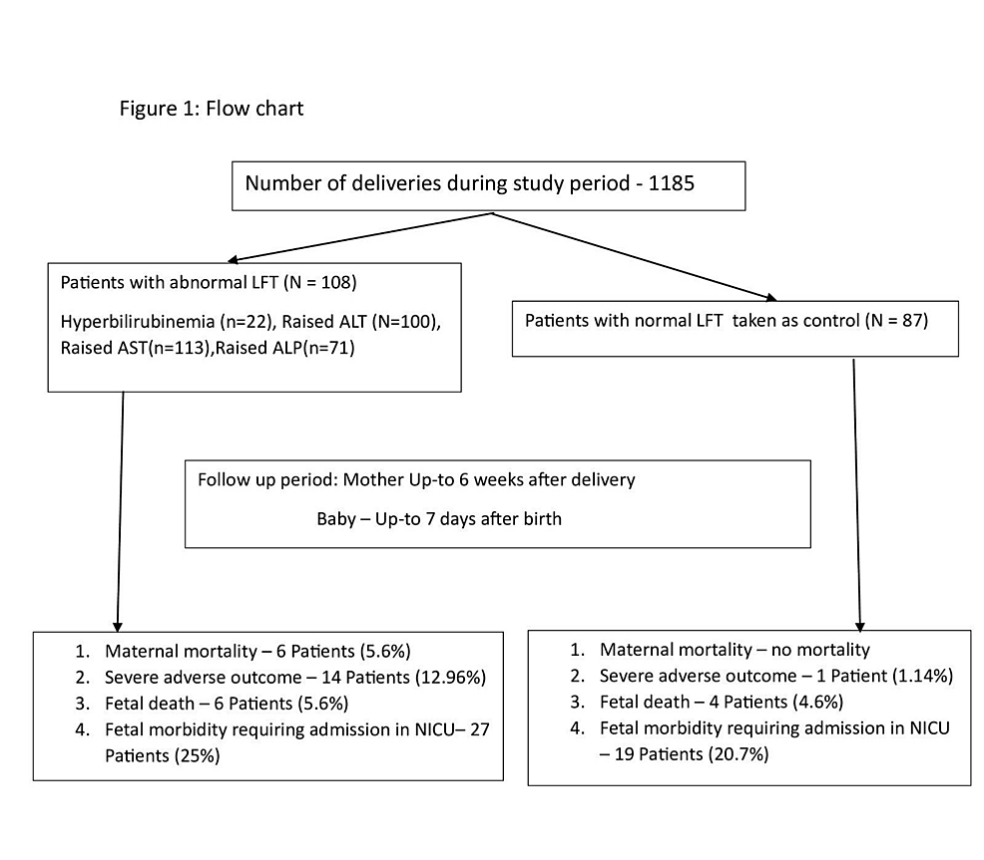
Flow chart of patients with abnormal LFTs. LFT: liver function test; AST: aspartate aminotransferase; ALT: alanine aminotransferase; ALP: alkaline phosphatase; NICU: neonatal intensive care unit.

Within our sample, 108 pregnant women had abnormal LFTs among 1185 deliveries, with a prevalence of 9.11%. Hyperbilirubinemia was seen in 22 patients, out of whom six had a bilirubin level >5 mg/dL. Hundred patients had raised ALT levels. ALT levels <2 times, two to five times, five to 10 times, and >10 times the upper normal level (UNL) were seen in 25, 42, 27, and six patients, respectively. Raised AST levels were seen in 113 patients, out of which 36, 39, 30, and eight patients had AST levels <2 times, two to five times, five to 10 times, and >10 times the UNL, respectively. ALP levels two to five times the UNL were seen in 67 patients. Only four patients had ALP levels >5 times the UNL. INRs of >1.5 were seen in seven patients. Feto-maternal outcomes of 87 pregnant women with normal LFTs were also analyzed for comparison (Figure 1).

Patients with abnormal LFTs had a similar average age of presentation (25.42 ± 4.33 vs. 25.2 ± 3.20 years) to that of patients with normal LFTs. Approximately half of the patients in both groups were primigravida (48.1% vs. 49.4%), and the presence of comorbidities (44.4% vs. 41.4%) was similar in both groups, as shown in Table 1. Major comorbidities or high-risk features associated with the abnormal LFT group were as follows: (a) hypothyroidism alone (four patients) and associated with bronchial asthma, recurrent pregnancy loss (RPL), ventricular septal defects, gestational diabetes mellitus (GDM), hypertensive disorder of pregnancy (HDP), and pleural effusion (one patient each); (b) isolated HDP (five patients) and associated with twin pregnancy with gallstone (one patient), sepsis with encephalopathy (one patient), and twin pregnancy (two patients); (c) twin pregnancy (one patient); (d) bad obstetrics history (three patients); (e) Rh negative (four patients); (f) premature rupture of membrane (PROM) (three patients); and (g) preterm PROM, TORCH infection, sepsis, RPL, rheumatic heart disease with mitral valve prolapse, placenta previa, polyhydramnios, oligohydramnios, paroxysmal nocturnal hemoglobinuria, extrahepatic portal vein obstruction, and ascites (one patient each). Patients with normal LFTs had the following major comorbidities or high-risk features: (a) HDP (nine patients); (b) beta-thalassemia (five patients); (c) hypothyroidism (four patients); (d) GDM (four patients); and (e) central placenta previa, dilated cardiomyopathy with RPL, rheumatic heart disease, RPL, HBsAg positive, and Rh negative (two patients each).

**Table 1:** Comparison of demographic and biochemical profile of pregnant patients with abnormal and normal LFTs. ALT: alanine aminotransferase; AST: aspartate aminotransferase; GDM: gestational diabetes mellitus, DM: diabetes mellitus; INR: international standardized ratio; SD: standard deviation; LFT: liver function test.

Parameter	Patients with abnormal LFTs (n=108)	Patients with normal LFTs (n=87)	p-value
Age in years (mean ± SD)	25.40 ± 4.33	25.20 ± 3.20	0.72
Gravida (G1: G2: G3, and above) (%)	48.1:26.9:16.7	49.4:35.6:12.6	
Presence of comorbidities	48 (44.4%)	36 (41.4%)	0.85 (0.80–0.90)
Previous cesarean section	10 (9.3%)	2 (2.3%)	0.08 (0.04–0.11)
DM including GDM	9 (8.3%)	4 (4.6%)	0.43 (0.36–0.50)
Hypothyroidism	13 (12%)	4 (4.6%)	0.07 (0.03–0.10)
Presence of anemia	23 (21.3%)	18 (20.7%)	1 (0.98–1.0)
Hemoglobin in g/dL (mean ± SD)	10.76 ± 1.61	10.91 ± 1.31	0.48
Leukocytes (×10^9^ cells/L) (mean ± SD)	10,826 ± 3265	10,623 ± 2811	0.64
Platelets (×10^9^ cells/L) (mean ± SD)	157 ± 55	183 ± 71	0.004
Median total bilirubin in mg/dL (range)	0.7 (0.13–20.83)	0.5 (0.12–1.0)	0.005
Direct bilirubin in mg/dL (range)	0.2 (0.04–11.83)	0.2 (0.04–0.54)	0.013
Median ALT in IU/mL (range)	124 (17–2576)	32 (15–39)	<0.001
Median AST in IU/mL (range)	136 (16–8188)	29 (15–38)	0.007
Alkaline phosphatase in IU/mL (mean ± SD)	298 ± 152	192 ± 58	<0.001
Protein in gm/dL (mean ± SD)	6.20 ± 0.78	6.29 ± 0.80	0.40
Albumin in gm/dL (mean ± SD)	3.37 ± 0.49	3.58 ± 0.62	0.009
INR	1.03 ± 0.54	0.85 ± 0.15	0.003
Fasting blood sugar in mg/dL (mean ± SD)	91 ± 25	81 ± 12	0.002
Creatinine in mg/dL (mean ± SD)	0.73 ± 0.20	0.65 ± 0.17	0.10
Uric acid in mg/dL (mean ± SD)	5.89 ± 1.71	5.58 ± 0.97	0.64

Most patients in either group did not have any symptoms. 60% of patients in the abnormal LFT group were asymptomatic at the time of the initial consultation, as compared to 85% of those in the normal LFT group. The most common presenting complaint in patients with abnormal LFT was pruritus (n=20, 18.5%), followed by jaundice (n=7, 6.5%) and leaking per vaginum (n=3, 2.8%). In patients with normal LFT, seven (8%) had pruritus, and two (2.3%) each had labor pain, leaking per vaginum, and convulsion. Hemoglobin and leucocyte count were similar in both groups; however, platelet count was significantly lower in the abnormal LFT group (157 ± 55×10^9^ cells/L vs. 183 ± 71×10^9^ cells/L, p-value=0.004). Obviously, significant differences in LFTs and their components were seen in the abnormal LFT group, as shown in Table 1. The mean gestational age at birth was 37.79 ± 1.71 weeks in patients with abnormal LFTs and 38.72 ± 1.55 weeks (p<0.001) in patients with normal LFTs.

Etiology of abnormal LFT

In our study, the etiology of abnormal LFT was elicited in 74% of cases. IHCP was found to be the most common cause of abnormal LFT, which accounted for 50% (54/108) of all cases, followed by HDP, including HELLP (7.4%), AFLP (4.6%), combined IHCP with HDP (3.6%), chronic liver disease (1.9%), gestational pruritus (1.9%), HDP with sepsis (0.9%), hepatitis E with antepartum eclampsia (0.9%), gallstone disease (0.9%), and paroxysmal nocturnal hemoglobinuria (0.9%). In 28/108 (26%) patients, the cause of the abnormal LFT could not be identified.

Mode of delivery

Among those in the abnormal LFT group, 50.9% (n=55) delivered vaginally, with 33 (60%) spontaneous and 22 (40%) induced deliveries, and 49.1% (n=53) delivered by cesarean section (CS). Twenty-seven pregnant women (51%) who underwent CS had spontaneous onset of labor, and induced delivery was seen in 26 patients (49%). Out of 48 patients in whom labor was induced, misoprostol, dinoprostone E2 gel, mifepristone, Foley’s catheter, and misoprostol combined with dinoprostone E2 gel were used in 19 (17.6%), 22 (20.4%), two (4.16%), two (4.16%), and three (6.25%) patients, respectively. Among the 53 patients who delivered via CS, 39 (73.6%) underwent emergency CS. The main indications of CS were fetal distress in 17 patients (43.58%), failed induction in 12 patients (30.76%), and previous CS with refusal for vaginal delivery in 10 (25.64%) patients.

Among the 87 patients in the normal LFT group, 63 (72.4%) delivered vaginally and 24 (27.6%) delivered by CS. Of the patients who delivered vaginally, spontaneous onset of labor was seen in 42 (66.6%) patients, and induction was done in 21 (33.3%) patients. Of the 24 patients who delivered via CS, 16 (66.6%) had spontaneous delivery, and 8 (33.3%) had induced delivery. Thus, 29 patients with normal LFTs underwent induction of labor, in which dinoprostone E2 gel was used in 20 patients and misoprostol in nine patients. Among the 24 patients who underwent CS, 18 were for emergency CS. The main indications of CS in patients with normal LFTs were fetal distress in 12 (66.6%) patients, previous CS with refusal for vaginal delivery in four (22.2%) patients, and failed induction in two (11.1%) patients.

Maternal outcome

There were six maternal deaths in the abnormal LFT group, whereas no maternal death was seen in the normal LFT group (p=0.03), as shown in Table 2. AFLP accounted for two maternal deaths. One patient each died of AFLP with HDP, sepsis, sepsis with hepatic encephalopathy, and jaundice with postpartum hemorrhage. All deaths occurred between 12 hours and five days after delivery (mean=2.1 days). Overall, 14 patients (12.96%) with abnormal LFTs suffered from severe adverse outcomes necessitating admission to the ICU. The main indications of admission to the ICU were AFLP (five patients), HDP with pleural effusion and sepsis (four patients), massive postpartum hemorrhage (three patients), antepartum eclampsia (one patient), and chronic liver disease (one patient). Only one patient in the normal LFT group required ICU admission due to antepartum eclampsia. The overall incidence of postpartum hemorrhage and the requirement for blood transfusion were not significantly different between groups.

**Table 2:** Comparison of maternal and fetal outcomes in patients with abnormal and normal LFTs. LFT: liver function test; NICU: neonatal intensive care unit; APGAR: appearance, pulse, grimace, activity, and respiration.

Parameter	Patients with abnormal LFTs (n=108)	Patients with normal LFTs (n=87)	p-value (95% confidence interval)
Delivery (spontaneous vs. induced)	60:48 (55.6%:44.4%)	58:29 (66.7%:33.3%)	0.13 (0.086–0.18)
Mode of delivery (vaginal vs. cesarean)	55:53 (50.9%:49.1%)	63:24 (72.4%:27.6%)	<0.001 (<0.001–0.015)
Emergency vs. elective cesarean	39:14	18:6	0.005 (0.001–0.015)
Presence of fetal morbidity	27 (25%)	22 (25.3%)	1 (0.98–1)
Presence of meconium	19 (17.6%)	8 (9.2%)	0.11 (0.07–0.16)
Development of postpartum hemorrhage	15 (13.9%)	8 (9.2%)	0.41 (0.34–0.48)
Blood transfusion requirement	17 (15.3%)	8 (9.2%)	0.23 (17–29)
Fetal mortality	6 (5.6%)	4 (4.6%)	1 (0.98–1)
Maternal mortality	6 (5.5%)	0	0.03 (0.003–0.048)
Maternal morbidity	14 (12.96%)	1 (1.1%)	<0.001 (<0.001–0.015)
NICU admission	23 (21.3%)	19 (21.8%)	1 (0.98–1)
Low APGAR	17 (15.7%)	9 (10.3%)	0.3 (0.23–0.36)

Fetal outcome

Fetal deaths were seen in six (5.6%) patients in the abnormal LFT group and four (4.6%) patients in the normal LFT group (p=1). In the abnormal LFT group, fetal deaths were seen in three patients with AFLP, in two patients with HDP with sepsis, and in one patient with IHCP; stillbirth was seen in three babies, and three had neonatal deaths. In the normal LFT group, two fetal deaths were seen in patients with antepartum eclampsia, and two occurred due to meconium aspiration syndrome.

Fetal morbidity was seen in 27 (25%) babies born to patients with abnormal LFT. 23 (21.3%) babies were admitted to the NICU. Indications of admission to the NICU included respiratory distress (11 patients), meconium aspiration (four patients), preterm baby (three patients), poor cry and low APGAR score and associated congenital abnormality (two patients), fetal cardiac arrhythmia (one patient), fever (one patient), and jaundice (one patient). In the normal LFT group, 19 (20.7%) neonates required admission to the NICU. The main indications for admission to the NICU included respiratory distress (12 patients), meconium aspiration syndrome (three patients), preterm babies (two patients), and low birth weight (two patients). Low APGAR score was seen in 15.7% and 10.3% of babies in the abnormal and normal LFT groups, respectively. The presence of meconium was similar in both groups. No significant differences were observed in the above-mentioned results when comparing the normal LFT group with the abnormal LFT group.

Predictors of maternal mortality

A total of six maternal deaths occurred out of 108 patients in the abnormal LFT group, and no maternal death occurred in the normal LFT group. Bivariate analysis showed a significant association of maternal death with low hemoglobin level, leukocytosis, thrombocytopenia, hyperbilirubinemia, raised transaminase and alkaline phosphatase levels, hypoproteinemia, hypoalbuminemia, coagulopathy, raised fasting blood sugar level, raised creatinine level, presence of fetal morbidity and mortality, postpartum hemorrhage, requirement of blood transfusion, prolonged hospital stay, and associated comorbidities, as shown in Table 3. Variables that were found to be significant by the bivariate analysis were taken for multivariate analysis, which showed that increased bilirubin and ALT were significantly correlated with maternal mortality.

**Table 3:** Bivariate analysis to determine the correlation of maternal mortality with several factors. SD: standard deviation; ALT: alanine aminotransferase; AST: aspartate aminotransferase; LFT: liver function test; INR: international standardized ratio; APGAR: appearance, pulse, grimace, activity, and respiration.

Parameter	Maternal mortality group (n=6)	Non-maternal mortality group (n=189)	p-value
Age in years (mean ± SD)	25.00 ± 2.09	25.29 ± 3.91	0.83
Presence of comorbidities	100%	57.8%	0.04
Hemoglobin in g/dL (mean ± SD)	8.75 ± 1.61	10.89 ± 1.45	<0.001
Leukocytes (×10^9^ cells/L) (mean ± SD)	14,675 ± 2731	10,629 ± 3015	0.001
Platelets (×10^9^ cells/L) (mean ± SD)	74 ± 24	171 ± 63	<0.001
Median total bilirubin in mg/dL (range)	8.5 (4.20–20.83)	0.6 (0.12–14.95)	<0.001
Median direct bilirubin in mg/dL (range)	5.20 (2.69–11.83)	0.2 (0.04–8.44)	<0.001
Median ALT in IU/mL (range)	340 (45–2576)	47 (15–2300)	<0.001
Median AST in IU/mL (range)	432 (42–8188)	45 (15–2000)	<0.001
Median alkaline phosphatase in IU/mL (range)	344 (247–923)	224 (65–846)	<0.001
Protein in gm/dL (mean ± SD)	4.71 ± 1.57	6.28 ± 0.71	<0.001
Albumin in gm/dL (mean ± SD)	2.68 ± 0.75	3.49 ± 0.54	<0.001
INR	2.68 ± 1.54	0.89 ± 0.17	<0.001
Abnormal LFT	100%	54%	0.026
Fasting blood sugar in mg/dL (mean ± SD)	106 ± 64	86 ± 18	0.02
Creatinine in mg/dL (mean ± SD)	1.92± 1.18	0.69 ± 0.22	0.001
Induced delivery	83.3%	59.8%	0.24
Gestational age at birth	37.60 ± 0.52	38.23 ± 1.74	0.37
Cesarean mode of delivery	33.3%	39.7%	0.75
Emergency cesarean	33.3%	10.2%	0.40
Presence of fetal morbidity	100%	33.9%	0.001
Presence of meconium	66.7%	12.2%	<0.001
Development of postpartum hemorrhage	100%	9%	<0.001
Fetal mortality	66%	3.2%	<0.001
Length of hospital stay in days (mean ± SD)	7.5 ± 8.8	1.03 ± 3.23	<0.001
Requirement of blood transfusion	100%	10.1%	<0.001
APGAR	83.3%	11.1%	<0.001

According to the bivariate analysis, thrombocytopenia, hyperbilirubinemia, elevated alkaline phosphatase, hypoproteinemia, coagulopathy, raised creatinine, gestational age at birth, presence of meconium, postpartum hemorrhage, maternal mortality, prolonged duration of hospital stay, blood transfusion requirement, and low APGAR score were significantly associated with fetal mortality, as shown in Table 4. However, only gestational age at birth, presence of meconium, APGAR score, maternal mortality, and raised alkaline phosphatase level were found to be significantly associated with fetal mortality in the multivariate analysis.

**Table 4:** Bivariate analysis to determine the correlation of fetal mortality with several factors. SD: standard deviation; ALT: alanine aminotransferase; AST: aspartate aminotransferase; LFT: liver function test; INR: international standardized ratio; APGAR: appearance, pulse, grimace, activity, and respiration.

Parameter	Fetal mortality group (n=10)	Non-fetal mortality group (n=183)	p-value
Age in years (mean ± SD)	26.30 ± 2.94	25.27 ± 3.92	0.41
Presence of comorbidities	100%	42.3%	0.008
Hemoglobin in g/dL (mean ± SD)	10.14 ± 1.80	10.86 ± 1.46	0.13
Leukocytes (×10^9^ cells/L) (mean ± SD)	11,650 ± 4621	10,714 ± 2971	0.34
Platelets (×10^9^ cells/L) (mean ± SD)	102 ± 30	172 ± 64	0.001
Median total bilirubin in mg/dL (range)	2.86 (0.5–20.83)	0.60 (0.12–14.95)	<0.001
Median ALT in IU/mL (range)	42 (17–526)	50 (15–2576)	0.61
Median AST in IU/mL (range)	48 (38–524)	49 (15–8188)	0.96
Median alkaline phosphatase in IU/mL (range)	403 ± 282	243 ±112	<0.001
Protein in gm/dL (mean ± SD)	5.40 ± 1.04	6.28 ± 0.75	0.001
Albumin in gm/dL (mean ± SD)	3.24 ± 0.88	3.47 ± 0.53	0.19
INR	1.75 ± 1.48	0.90 ± 0.22	<0.001
Abnormal LFT	60%	55.2%	0.76
Fasting blood sugar in mg/dL (mean ± SD)	78 ± 19	87 ± 21	0.19
Creatinine in mg/dL (mean ± SD)	1.10 ± 0.39	0.70 ± 0.35	0.001
Induced delivery	40%	38.8%	0.94
Gestational age at birth	36.33 ± 1.31	38.30 ± 1.67	<0.001
Cesarean mode of delivery	30%	39.9%	0.53
Presence of fetal morbidity	100%	33.3%	0.003
Presence of meconium	90%	9.8%	<0.001
Development of postpartum hemorrhage	50%	9.8%	<0.001
Maternal mortality	40%	1.1%	<0.001
Length of hospital stay in days (mean ± SD)	4.90 ± 7.30	0.98 ± 3.14	0.001
Requirement of blood transfusion	50%	10.9%	<0.001
APGAR	30%	89.6%	<0.001

## Discussion

The prevalence of abnormal LFTs was 9.11% within the study sample, with IHCP being the most common cause. Most Indian studies reported a prevalence of abnormal LFTs ranging from 0.72% to 6.7% [2,13]. The relatively higher prevalence of abnormal LFTs in our study may be due to referral bias, as our center mostly takes referrals from multiple other centers. The higher prevalence may also be due to different inclusion criteria, as some studies only included jaundiced patients [2], some included only admitted patients, and others included admitted patients as well as outpatients. Finally, we included only third-trimester patients, which may have contributed to the higher prevalence of abnormal LFTs in our sample.

In our study, the most common cause of abnormal LFTs was IHCP (50%), followed by HDP, including HELLP syndrome. The etiologies of abnormal LFTs vary across different studies. Recently, a systematic review showed that HDP (mean=36.0%, median=31.4%, range=3.6%-83.8%), viral hepatitis (mean=34.1%, median=35.5%, range=5.1%-61.8%), and IHCP (mean=16.9%, median=15.0%, range=1.2%-54.9%) were the three most common causes of liver dysfunction in pregnancy [5]. A relatively higher incidence of IHCP was seen in studies by Dsouza et al. (54.9%) and Kohli et al. (35.6%) where the diagnosis of IHCP was made on clinical grounds, similar to our study [13,14]. Bile acid estimation was not done in the majority of patients in the current study due to economic constraints.

In the abnormal LFT group, a significantly higher number of patients underwent CS (49.1%) as compared to patients in the normal LFT group (27.6%). The rate of CS in the abnormal LFT group in our study was similar to that reported by Kohli et al. at 41% of cases [13]. In a study by Suresh et al., 76% of patients underwent CS [15], whereas CS was done in only 24.2% of cases in a prospective study by Kishore et al. [2]. This variability in CS rate may be due to etiological differences among patients with abnormal LFTs as well as different protocols for CS adopted by different institutes.

Our results showed a maternal mortality rate of 5.5% in our sample, which was similar to studies by Malhotra et al. (6.24%) and Bhalla et al. (5.9%) [16,17]. Maternal mortality varies widely across different studies, ranging from 1.37% to 39.3% [12,13]. Generally, studies with viral hepatitis or HDP as the main etiology are associated with higher mortality than studies with IHCP as the main etiology. In a systematic review of 21 studies from India, the mean maternal mortality rate was shown to be 12.6% in patients with abnormal LFTs [5]. Half of the maternal deaths in our study were caused by AFLP alone (33.3%) or associated with HDP (16.6%), and sepsis with or without encephalopathy was responsible for 33.3% of maternal deaths. Viral hepatitis [18], HDP [13,15], AFLP [19,20], tropical hepatitis [21], sepsis, and encephalopathy [12,22] were the principal causes of maternal mortality, as described in various other Indian studies. The perinatal mortality rate was 5.56% in our study, which was similar to that reported by Dsouza et al. at 5.7% of cases [14]. Similar rates of fetal death were also observed in other studies [3,23].

Higher fetal death rates were seen in studies by Suresh et al. (20%) and Mishra et al. (41.25%) [15,20]. In other studies, a higher number of fetal adverse events were encountered in patients with HDP, including HELLP syndrome, sepsis, hepatitis E, and chronic liver disease. Very few fetal deaths were seen in patients with IHCP. Our study found serum bilirubin and ALT level to be significantly associated with maternal mortality, whereas preterm birth, presence of meconium, low APGAR score, associated maternal mortality, and raised creatinine and alkaline phosphatase levels were found to be significantly associated with fetal mortality according to the multivariate analysis. In a prospective observational study by Kishore et al. [2], higher total serum bilirubin, higher serum AST, anemia, and abnormal INR were significantly correlated with maternal mortality. Suresh et al. also showed that maternal anemia, thrombocytopenia, and coagulopathy were significantly correlated with adverse fetal outcomes, whereas thrombocytopenia and hyperbilirubinemia were significantly correlated with adverse maternal outcomes. Serum bilirubin performed better than INR as a predictor of both maternal and fetal outcomes in other studies [15,17].

Our study did have certain limitations. The main limitation was that only third-trimester patients were included in this study, and, therefore, causes of abnormal LFT in second- and first-trimester patients were not included. Furthermore, as our center is a referral center, the possibility of referral bias could not be ruled out, limiting the generalizability of our results. Another limitation of the study was that patients attending an antenatal clinic with an asymptomatic elevation of LFT could not be tested. Despite its limitations, this study is the first in India to compare the feto-maternal outcomes of patients with abnormal LFTs with those of normal LFTs. It also provides important comparative information on feto-maternal outcomes in patients with normal and abnormal LFTs from the eastern part of India.

## Conclusions

Patients with abnormal LFTs were significantly associated with maternal mortality and morbidity, which were both significantly higher in patients with sepsis-induced liver dysfunction. However, the liver dysfunction associated with IHCP had a relatively uneventful outcome. The fetal outcomes in patients with abnormal and normal LFTs were similar. Hyperbilirubinemia and raised ALT were significant predictors of maternal mortality. Watchful monitoring and timely intervention are required to reduce adverse maternal outcomes in pregnant women with liver dysfunction due to conditions such as AFLP, sepsis, and HDP.

## References

[1] Guarino M, Cossiga V, Morisco F (2020). The interpretation of liver function tests in pregnancy. Best Pract Res Clin Gastroenterol.

[2] Kishore R, Thakur N, Tuwani M (2021). Evaluation of maternal and fetal outcome in pregnancies complicated by jaundice-an observational study. Int J Reprod Contracept Obstet Gynecol.

[3] Sumangli PK, Kurian S (2017). Study of abnormal liver function tests in pregnancy in a tertiary centre in North Kerala. Int J Med Res Sci.

[4] Joshi D, James A, Quaglia A, Westbrook RH, Heneghan MA (2010). Liver disease in pregnancy. Lancet.

[5] Ahmed A, Saxena S, Pandey A, Mishra P, Azim A (2022). Analysis of causes of hepatic dysfunction in obstetric patients in India: a systematic review. Indian J Crit Care Med.

[6] Bhide A, Arunkumaran S, Damania KR (2020). Arias' Practical Guide to High-Risk Pregnancy & Delivery: A South Asian Perspective, 5th ed.

[7] Solanke D, Rathi C, Pandey V, Patil M, Phadke A, Sawant P (2016). Etiology, clinical profile, and outcome of liver disease in pregnancy with predictors of maternal mortality: a prospective study from Western India. Indian J Gastroenterol.

[8] Dutta DC (2018). DC Dutta's Textbook of Obstetrics. Textbook of obstetrics.9th edition, page.

[9] (2021). Ministry of Health and Family Welfare. Maternal mortality rate (MMR). http://pib.gov.in/PressReleaseIframePage.aspx.

[10] Tiwari R, Kushwaha P, Meravi A (2020). Analytical study to determine the impact of jaundice in pregnancy on maternal and perinatal outcome. Adv Hum Biol.

[11] Padh JP, Shah SR, Vyas RC, Parikh PM (2019). A clinical study on fetomaternal outcome in jaundice with pregnancy. Int J Reprod Contracept Obstet Gynecol.

[12] Agrawal M, Bhanu M, Sankhwar P, Deo S, Jaiswar S (2019). A study of spectrum and fetomaternal outcome of jaundice in pregnant women: an experience from a tertiary referral centre of North India. Int J Reprod Contracept Obstet Gynecol.

[13] Kohli UA, Seth A, Singh S, Mishra R (2017). Liver ailments in pregnancy: our experience. Int J Reprod Contracept Obstet Gynecol.

[14] Dsouza AS, Gupta G, Katumalla FS, Goyal S (2015). Maternal and fetal outcome in liver diseases of pregnancy: a tertiary hospital experience. Int J Sci Res Publ.

[15] Suresh I, Vijaykumar TR, Nandeesh HP (2017). Predictors of fetal and maternal outcome in the crucible of hepatic dysfunction during pregnancy. Gastroenterol Res.

[16] Malhotra V, Priyanka Narang P, Nanda S, Bhuria V, Malhotra P (2018). Maternal and perinatal outcome in pregnancies complicated by jaundice. Int J Enhanced Res Med Dent Care.

[17] Bhalla S, Bhatti SG, Kumar S, Kaur P (2019). Predictors of feto-maternal outcome in pregnancies complicated by hepatic dysfunction: observational study in a tertiary care hospital in Punjab. Pan Asian J Obstet Gynecol.

[18] Sharma S, Aherwar R, Jawade S (2016). Maternal and fetal outcome in jaundice complicating pregnancy: a prospective study. Int J Reprod Contracept Obstet Gynecol.

[19] Krishnamoorthy J, Murugesan A (2016). Jaundice during pregnancy: maternal and fetal outcome. Int J Reprod Contracept Obstet Gynecol.

[20] Mishra N, Mishra VN, Thakur P (2016). Study of abnormal liver function test during pregnancy in a tertiary care hospital in Chhattisgarh. J Obstet Gynaecol India.

[21] Mitta P, Rao SV (2016). Fetomaternal outcome in jaundice complicating pregnancy. J Dent Med Sci.

[22] Tiwari A, Aditya V, Srivastava R, Gupta G (2017). A study of spectrum and outcome of liver diseases in pregnant women at BRD medical college. Int J Reprod Contracept Obstet Gynecol.

[23] Tripathi R, Brahmane M, Jain SB (2020). Effects of abnormal liver function test on maternal and perinatal outcome in pregnancy: observational study. Int J Reprod Contracept Obstet Gynecol.

